# Molecular Mechanisms Affecting Statin Pharmacokinetics after Bariatric Surgery

**DOI:** 10.3390/ijms251910375

**Published:** 2024-09-26

**Authors:** Matea Petrinović, Domagoj Majetić, Miro Bakula, Ivan Pećin, Daniela Fabris-Vitković, Marin Deškin, Deša Tešanović Perković, Maja Bakula, Marina Gradišer, Ines Bilić Ćurčić, Silvija Canecki-Varžić

**Affiliations:** 1 Faculty of Medicine Osijek, Josip Juraj Strossmayer University of Osijek, 31000 Osijek, Croatia; mateapetrinovic@gmail.com (M.P.); dmajetic8@gmail.com (D.M.);; 2 Polyclinic Slavonija, 31000 Osijek, Croatia; 3 The Clinic for Internal Diseases, Department for Gastroenterology and Hepatology, Clinical Hospital Centre Osijek, 31000 Osijek, Croatia; 4 Department of Internal Medicine, Division of Endocrinology, Diabetes and Metabolic Diseases, Sveti Duh University Hospital, 10000 Zagreb, Croatia; mbakula@kbsd.hr; 5 Department of Internal Medicine, Unit for Metabolic Diseases, University Hospital Center Zagreb, 10000 Zagreb, Croatia; 6 School of Medicine, University of Zagreb, 10000 Zagreb, Croatia; 7 Division of Endocrinology and Diabetes, General Hospital Pula, 52100 Pula, Croatia; 8 Special Hospital Agram, 10000 Zagreb, Croatia; 9 Vuk Vrhovac University Clinic for Diabetes and Metabolism, Merkur University Hospital, 10000 Zagreb, Croatia; 10 Internal Medicine Department, County Hospital Čakovec, 40000 Čakovec, Croatia; 11 School of Medicine, University of Split, 21000 Split, Croatia; 12 Clinic for Internal Diseases, Department for Endocrinology and Diabetes, Clinical Hospital Centre Osijek, 31000 Osijek, Croatia; 13 Faculty of Dental Medicine and Health, Josip Juraj Strossmayer University of Osijek, 31000 Osijek, Croatia

**Keywords:** bariatric surgery, dyslipidemia, hypolipidemic agents, medication management, obesity, inhibitors, hydroxymethylglutaryl-CoA reductase

## Abstract

According to recent data, one in eight people in the world struggle with obesity. Obesity management is increasingly dependent on bariatric surgical interventions, as the combination of lifestyle modifications and pharmacotherapy could have a modest long-term effect. Surgery is recommended only for individuals whose body mass index (BMI) ≥ 40 kg/m^2^ and ≥ 35 kg/m^2^ in the presence of weight-related comorbidities. The most commonly performed procedures are sleeve gastrectomy and roux-en-Y gastric bypass. Pharmacokinetic and pharmacodynamic alterations occur as a result of the anatomical and physiological changes caused by surgery, which further differ depending on physicochemical drug factors and factors related to the dosage form. The following modifications are distinguished based on the type of bariatric surgery performed. Most bariatric patients have accompanying comorbidities, including dyslipidemia treated with hydroxymethylglutaryl-coenzyme A (HMG-CoA) reductase inhibitors or statins. Significant improvements in the lipid profile are observed early in the postoperative period. The data reported in this review on statin pharmacokinetic alterations have demonstrated substantial inter- and intravariability, making it difficult to adopt clear guidelines. Based on the current literature review, reducing the statin dose to the lowest effective with continuous monitoring is considered an optimal approach in clinical practice.

## 1. Introduction

Over the last 30 years, obesity has become a global health problem. The prevalence of obesity has more than doubled among adults and even quadrupled in the population of children over the last two decades [1]. The prevalence of overweight and obesity varies by region, but a growing trend is demonstrated in both highly-industrialized and less-industrialized countries. According to the last update from the World Health Organization (WHO), one in eight people in the world struggle with obesity [2].

Obesity is defined as an abnormal, excessive accumulation of adipose tissue, and the most widely used formula to define overweight and obesity is body mass index (BMI). BMI is an indirect measure of anthropometrics that includes body weight and height. Individuals are classified as overweight (pre-obese) if their BMI is between 25 and 29.9 kg/m^2^, and obese if greater than or equal to 30 kg/m^2^, with three different categories: moderately, severely, and very severely obese [3]. BMI is often challenged as a measure of adiposity and health outcomes at the individual level because the percent fat and risk for any given BMI can be highly variable and secondary to the patient’s sex, age, race/ethnicity, cardiovascular fitness level, and many other factors. Alternative anthropometric measurement indices include waist circumference, waist/hip ratio, and waist/height ratio, which describe the distribution of body fat more precisely than BMI [4]. Obesity relates to an increased risk of serious comorbidities, such as hypertension, insulin resistance, and type 2 diabetes mellitus (T2DM), and is associated with a 13- to 18-fold increased risk, compared to normal-weight individuals, of hyperlipidemia, atherosclerosis, coronary artery disease, depression, and malignant tumors [5,6]. In terms of the effect of obesity on overall mortality, an increase in BMI of 5 kg/m^2^ is associated with a 30% higher mortality risk [7]. 

The management of obesity requires a combination of lifestyle modifications and pharmacotherapy, or bariatric surgery [8]. Even though lifestyle interventions remain the basis of initial treatment, adherence is often inadequate, and long-term success is modest precisely due to the complexity and multifactorial nature of obesity, including environmental, social, and genetic factors [9]. Five medications are available on the market in the United States (US) and Europe for weight management: naltrexone/bupropion, liraglutide, semaglutide, orlistat, and tirzepatide. The Food and Drug Administration (FDA) also approved lorcaserin and phentermine/topiramate on the US market [9,10]. These drugs are reserved for patients with a BMI of ≥30 kg/m^2^ and a BMI of ≥27 kg/m^2^ who have at least one obesity-related comorbidity and are the next step in obesity treatment for patients who have difficulties maintaining weight loss over a longer period [5,10,11]. All the above-mentioned conservative weight loss interventions could cause lost weight to be regained, especially when antiobesity medications are continued over a year, and even more when the pharmacotherapy treatment ends [12].

Due to the side effects and limited effectiveness of conservative measures, there has been a considerable increase in the use of bariatric surgery in clinical practice to reduce the body mass of morbidly obese patients. Bariatric surgery is currently superior in its effects regarding both weight loss and its maintenance, as well as accompanying comorbidities [13].

In this narrative review article, we aimed to evaluate the available evidence about changes in the pharmacokinetic profile of statins in obese subjects after bariatric surgery.

## 2. Methods

The National Library of Medicine’s PubMed, Scopus, and Web of Science were systematically searched to find relevant studies. Search terms included obesity, obese, bariatric surgery, bariatrics, gastric bypass, RYGB, and roux-en-Y, combined with related terms such as statin pharmacokinetics, drug absorption, medication adjustment, and medication management. The number of relevant studies analyzed in this article was 29.

The search was limited to studies on human adults and publications written in the English language. Due to the small number of studies regarding the pharmacokinetic changes of statins after bariatrics in recent years, the time criterion has not been applied.

## 3. Results and Discussion

### 3.1. Bariatric Surgery

In the past decade, about half a million bariatric procedures were performed annually worldwide [14]. Surgery is indicated for patients with BMI ≥ 40 kg/m^2^ and ≥35 kg/m^2^ in the presence of weight-related comorbidities [15]. Bariatric surgical procedures are classified into three main categories: restrictive (laparoscopic gastric banding, gastrectomy), malabsorptive (jejunoileal bypass), and integrated restrictive/malabsorptive procedures (roux-en-Y gastric bypass; biliopancreatic diversion) [16,17]. The most common surgical procedures include sleeve gastrectomy (SG, 76.5%), roux-en-Y gastric bypass (RYGB, 21.2%), laparoscopic adjustable gastric banding (LAGB, 1.3%), and biliopancreatic diversion with duodenal switch (BPD-DS, 1%), supported by a review published by the American Society for Metabolic and Bariatric Surgery in 2020 [18,19].

In the SG procedure, a staple line is placed along the greater curvature of the stomach, followed by the removal of approximately 80% of the lateral aspect of the stomach, so this surgical procedure does not require a gastrointestinal anastomosis or intestinal bypass. A reduction in stomach size with sleeve resection restricts distention and increases the patient’s satiety [20]. SG is also considered a metabolic procedure, attributed to the decreasing serum levels of ghrelin, a hormone produced mainly by programmed cell death protein 1 (P/D1) cells that stimulates hunger [21]. SG is the most prevalent method of operative treatment nowadays, as it is safe, effective, and less technically challenging than laparoscopic RYGB [22]. However, the literature shows a 75.6% weight regain within six years post-SG, related to the mainly restrictive mechanism [23,24].

Until 2013, RYGB represented the golden standard in the United States for weight loss in morbid obesity. RYGB showed the largest sustained weight loss, with further improvements in comorbidities, especially metabolic disorders such as T2DM, quality of life, and mortality rates [22,24,25]. In RYGB, the proximal portion of the stomach is reattached to a more central part of the small intestine, creating a gastric sac with a capacity of 20–30 mL, and bypassing the duodenum and 50–70 cm of the jejunum. The continuity of the gastrointestinal tract is restored by creating a Roux-en-Y branch of the gastrointestinal tract in the jejunum, which requires gastrojejunal anastomosis, so this procedure is much more demanding than SG and has a slightly higher rate of complications [10,26,27]. Weight loss of about 57–67% is the result of both diet restriction and decreased absorption due to short-circuiting and hormonal changes [28].

LAGB was first performed in 1983 and reached its peak in 2008. However, it has turned into the third most common procedure over the last few years [23,29]. The benefit of a band or ring around the stomach starts in the mid- and long-term post-surgery period, when the dilatation of the gastric pouch becomes responsible for weight regain and the band contributes to early satiety [30]. This intervention is purely restrictive and leads patients to change their eating behavior. However, the effectiveness of this procedure declines over time as patients adjust their eating habits [31]. Data from the literature regarding this procedure are contradictory; initially, it was considered to be a safe, quick, minimally invasive procedure, but despite upgrades in surgical strategy, it has been proven to have significant complications that require reoperation with band disposal and an almost 40% failure rate over the five-year follow-up [32,33].

The fourth most common bariatric surgical procedure is BPD-DS. This is also a restrictive and malabsorptive procedure in which the volume of the gastric remnant is reduced (by approximately one-third of normal) and the biliopancreatic limb is reconnected to the intestine 50–100 cm proximal to the ileocecal valve. Consequently, ingested substances are exposed to bile and pancreatic juices only in the very last part of the ileum [34,35]. However, BPD-DS represents only 1% of the bariatric surgeries performed worldwide. The reasons for this finding include increased technical complexity, high complication and mortality rates reported in the literature, and an increased risk of protein malnutrition [36,37].

### 3.2. The Repercussion of Bariatric Surgery on Drug Pharmacokinetics

#### 3.2.1. Anatomical and Physiological Alterations Following Bariatric Surgery with Potential Implications Regarding Drug Pharmacokinetics

Bariatric surgery procedures as a consequence of anatomical and physiological modifications cause pharmacokinetic quality changes after oral drug administration (Table 1). Restrictive techniques reduce gastric natural capacity and therefore limit the net volume of food one can ingest at one time, while malabsorptive techniques mainly alter the physiological digestive process by shortening the functional surface area of the small intestine, leading to restricted macro- and micronutrient absorption. Procedures with combined restrictive and malabsorptive surgical components aim to implement both [17,38].

Multiple pharmacokinetic changes are occurring and, along with the weight loss, could be responsible for a poor therapeutic response to medications. The rate and extent of drug absorption from the gastrointestinal tract depend on very complex biopharmaceutical processes and are modified by many factors [39]. Medication factors to consider post-surgery can be divided into two categories: physicochemical factors of the drug (stability, diffusivity, solubility, polarity, ionization, dissolution, and particle size) and factors related to the dosage form (e.g., tablet, capsule, solution, suspension, or emulsion), whereas physiological factors include gastric emptying, gastric and intestinal pH, small intestinal transit time, and area of mucosal exposure [42]. An increased rate of absorption does not necessarily lead to an increased extent of absorption, evaluated by the area under the curve (AUC). The AUC of orally administered drugs depends on the extent of absorption and elimination, as both are affected by the modification induced by surgery and the associated weight loss [39].

After restrictive bariatric surgery, gastric volume and stomach surface area are significantly reduced, and the emptying rate of the stomach is altered [43,44]. Gastric emptying time can serve as the rate-limiting step for highly permeable and highly soluble drugs because absorption from the stomach is inevitably low. Although gastric emptying time is modified after the surgery, this would not be expected to change the overall extent of drug absorption by itself because the area under the curve is primarily affected by the small intestinal absorptive area [16]. Gastric pH is increased because the number of acid-producing parietal cells is reduced, leading to poor dissolution of pH-dependent acid-soluble drugs [45]. The above-mentioned alterations in the physiology of the stomach are still not those that significantly affect drug absorption, as the small intestine is considered a major site of drug absorption.

#### 3.2.2. Molecular Mechanisms Affecting Drug Pharmacokinetics after Bariatric Surgery

**Figure 1:**
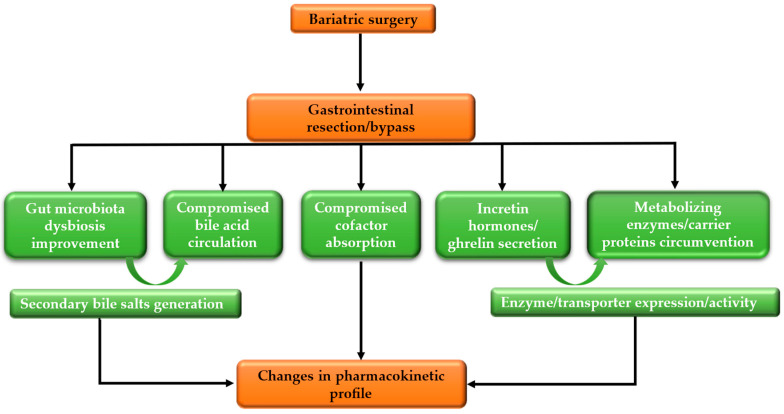
Molecular changes affecting drug pharmacokinetics after bariatric surgery.

Along the small intestine, changes that occur at the cellular level produce considerable adjustments in the pharmacokinetics of drugs, as illustrated in Figure 1. The upper small intestine’s normal milieu includes surfactants derived from pancreatic secretion [42]. Some procedures, such as RYGB, affect the enterohepatic circulation of bile acid by resecting or bypassing the duodenum.

A study by De Giorgi et al. showed that, as well as the alterations in bile circulation and the increase in the total amount of bile acid flux, the peaks in the plasma concentration of bile acids were higher and occurred earlier after a meal compared to weight-matched control patients [46,47]. Medications that interact with bile salts as endogenous surfactants to improve their solubility—particularly drugs with lipophilic properties (e.g., selective serotonin receptor inhibitors and thyroxin)—may be deficiently solubilized [48,49]. In addition, certain surgical methods circumvent the great portion of the small intestine in which a variety of transporters and metabolizing enzymes are expressed. This bypass allows medications to be transported directly into the jejunum and ileum, a part of the intestine that is, compared to the duodenum, less metabolically active, resulting in altered oral bioavailability [31,44].

The dominant drug-metabolizing enzymes are cytochrome P450 (CYP), uridine diphosphate glucuronosyltransferase (UGT), sulfotransferases, and glutathione S-transferases, the most numerous of which are the CYP3A subfamily. About half of the most prescribed drugs are substrates for CYP3A4, expressed at lower levels in the duodenum, rising in the jejunum and decreasing towards the ileum, so impairment in oral bioavailability is expected after such a procedure [50].

Similar to enzymes, the expression of carrier proteins is higher in the proximal part of the small intestine and decreases with progression to the more distal area [51]. The level of expression of the carrier protein is a major determinant of the drug’s permeability in different directions, including both influx transporters and efflux pumps [52]. The efflux transporter P-glycoprotein (P-gp) is another important protein that modifies drug bioavailability. The expression of P-gp, along with CYP enzymes, shows high interindividual variability and varies over the length of the intestinal tract [53]. On the other hand, the activity of CYP enzymes in the liver was found to be more prominent post-surgery, leading to the conclusion that it can be inversely correlated to body weight [45,54,55]. Therefore, drugs that are metabolized by CYP3A4 may be metabolized more quickly, leading to decreased plasma concentrations and potential therapeutic failure. As a result, an increase in the dose of these drugs may be needed to achieve the desired therapeutic levels. However, due to changes in other physiological parameters, such as liver flow (approximately 20% lower after bariatric surgery), this normalization may not necessarily translate into a higher clearance of drugs that are hepatically metabolized by CYP3A [54,56].

In addition, nutritional deficiencies due to compromised absorption of vitamins and minerals that are cofactors for various metabolic enzymes potentially impact drug metabolism. Iron is mainly absorbed in the duodenum and proximal jejunum, and iron deficiency can especially affect the function of CYP enzymes, as it is an essential part of the heme group. B group vitamins, such as B1, B2, B3, and B6, as well as vitamin C, work as coenzymes or precursors of flavin and nicotinamide nucleotides, and as such could reduce the activity of CYP enzymes and affect drug clearance [31].

Altered levels of incretin hormones and ghrelin can influence metabolic pathways and drug responses. Incretin hormones, primarily glucagon-like peptide-1 (GLP-1) and glucose-dependent insulinotropic polypeptide (GIP), regulate glucose homeostasis, aiming to reduce food intake. Ghrelin, on the other hand, is a gastric-derived hunger hormone predominantly produced in the stomach and plays a significant role in regulating appetite and energy balance [57]. Many studies have reported that bariatric surgery enhances incretin hormone secretion, while reports regarding ghrelin secretion have been ambiguous, most likely due to technical differences between the bariatric procedures concerning vagotomies. [57,58,59]. Though less direct, hormonal changes might substantially influence absorption rate and drug dissolution, primarily through their effects on gastric motility and emptying, as well as on the modulation of intestinal transit time. Incretin hormones and ghrelin also modulate the expression and activity of drug transporters and metabolizing enzymes.

Ghrelin’s effects on increasing fat storage and distribution can impact the volume of distribution for lipophilic drugs. Features of body composition and disposition of medications cannot be considered identical between individuals who were obese before regulating their body weight and those who were never obese [60]. Total body weight is lower, and the change in body composition (the percentage of adipose tissue versus lean mass) leads to drug distribution alterations. In the context of bariatric surgery, the physiological and pharmacokinetic consequences arise from the surgical procedure itself and are integrated with the consequences of subsequent weight loss.

Lastly, the gut microbiota, with approximately three trillion bacteria, is involved in host metabolism regulation. Gut microbiota dysbiosis is a main characteristic of obesity, associated with decreased microbial gene richness that further worsens the severity of obesity, pro-inflammatory response, and metabolic regulation [61,62]. Manipulation of the intestinal anatomy by bariatric surgery, either directly or indirectly through weight loss, influences the microbiota stasis and diversity within the gut. Direct influence involves changes in the pH, GI motility, and composition of the bile salts [46]. Primary bile salts produced in the liver are dehydroxylated by the microbiome in the intestinal tract to generate secondary bile acids, both primarily increased after surgery, with a strong correlation with triglyceridemia reduction [63,64]. Altered bile acid composition, gut permeability, metabolite production, and microbial enzyme activity may remodel drug efficacy or toxicity profile due to changes in gut microbiota.

#### 3.2.3. General Guidelines on Drug Management after Bariatric Surgery

A study design is an important criterion for assessing data. A combination of longitudinal and cohort designs probably constitutes the most reasonable overall research strategy [60]. A great number of studies are based on comparisons of cohorts of (formerly) obese individuals with parallel cohorts of normal-weight controls. Methodologically, the limiting factor is that the control groups consisted of nonsurgical patients who had never been obese, creating a potentially confounding problem. Consequently, it is necessary to manage medication regimens after surgery. Such guidelines cannot be accurately created and applied in general to all patients, assuming that they depend on several surgical-procedure-specific and patient-specific factors. High inter- and intravariability among patients regarding changes in absorption kinetics explain why every patient demands an individual approach in the treatment process [16].

These drug absorption alterations can also be temporary or permanent. In these cases, close monitoring of the patient and long-term medical follow-up are required to make adjustments if suboptimal medication absorption or potential drug toxicity are suspected.

The European Association for the Study of Obesity has highlighted that the “potential effects and consequences that any bariatric procedure may have on the absorption and action of drugs should be carefully studied before surgery” [65]. Based on the previously described drug disposition changes following bariatrics, a few general approaches have been adopted [40].

The first recommendation concerns the drug formulation itself: solid dosage forms should be substituted with liquid forms or dissolvable or crushable tablets. In cases where only a solid dosage form of the drug exists, patients should be instructed to crush or open it and disperse the powder in liquid before ingestion [41]. Extended-release formulations should be switched to immediate-release formulations [66]. Since the duodenum and the proximal jejunum are bypassed in RYGB and the time for passage through the intestine is shortened, inadequate transit time may prevent the full dissolution and absorption of poorly soluble drugs or extended-release drug formulations [38,67].

Second, the intake of medications with first-pass metabolism should be minimized as it is decreased, as well as the intake of drug groups that irritate the upper part of the gastrointestinal tract or delay the healing process, e.g., corticosteroids, oral bisphosphonates, and nonsteroidal anti-inflammatory drugs [66,67]. A parenteral drug administration (e.g., intravenous, intramuscular, subcutaneous, vaginal, rectal, or nasal dosage forms) is preferred to prevent sub- or supratherapeutic effects [49].

Apart from these general guidelines, each group of drugs shows a different pattern of pharmacokinetic changes after bariatric surgery. Specifically, in this review, the emphasis is on statins, given that these medications are often used in this group of patients due to accompanying dyslipidemia, and that the lipid panel changes dynamically after the bariatric procedure.

### 3.3. Influence of Bariatric Procedures on Lipid Profile

A large portion of obese patients have lipid–lipoprotein abnormalities as an accompanying comorbidity. The atherogenic dyslipidemia associated with severe obesity is characterized by elevated fasting and postprandial triglyceride (TG) levels, low high-density lipoprotein cholesterol (HDL-c) concentrations, and an increased proportion of low-density lipoprotein cholesterol (LDL-c) [68]. This lipid profile is extremely atherogenic and is associated with an elevated cardiovascular risk (CVR). In a study conducted by Bays et al., 60% of obese individuals were diagnosed with dyslipidemia and identified as candidates for surgical treatment [69]. Significant improvements in anthropometric and metabolic parameters, including the lipid–lipoprotein profile following bariatric surgery, occur early in the postoperative period.

An article published by do Nascimento et al. compared observed changes in the lipid profile after certain subtypes of bariatric surgery procedures [70]. Patients undergoing RYGB had better values of total cholesterol, LDL-c, HDL-c, and TG levels two years after surgery. On the other hand, patients submitted to SG showed a slight advance in TG level but none in LDL levels [70,71,72]. These findings coincide with the results of other similar studies, including Buchwald’s meta-analysis of 22,000 patients, 70% of whom showed long-lasting improvements in their lipid–lipoprotein profile after RYGB [73].

In general, malabsorptive procedures obtain a higher decrease in total cholesterol (TC) and LDL during follow-up and consequently improve CVR [74]. RYGB, which is both a malabsorptive and a restrictive procedure, attains the biggest reduction in plasma lipid levels. Among purely restrictive surgical subtypes, SG is superior only in terms of a significant increase in levels of HDL-c, reaching normal levels in 58.1% of post-SG male patients versus 39.5% of post-RYGB male patients [75]. Data for post-gastric bypass and post-SG patients are comparable for changes in lipid profile with the previously mentioned advantage of SG considering HDL-c [76]. The response within a five-year follow-up after these subtypes of bariatric surgery is usually not significantly different from that of nonsurgical control patients [77]. To summarize, the type of bariatric surgery procedure performed was declared the strongest independent predictor for all lipid level improvements or remissions, apart from weight loss, with the supremacy of techniques with a certain malabsorptive component. This may be because malabsorptive surgery decreases cholesterol absorption, thus producing a decrease in LDL-c and TC.

However, this cholesterol absorption is not modified with restrictive techniques, so LDL-c tends to increase or not decrease as much with non-malabsorptive surgeries [77,78]. Not all patients manage to regulate their lipid profile by body weight reduction alone. As a result of all the previously mentioned physiological changes in the body that follow bariatric surgery, a question arises about the types of medications and dosages to regulate the lipid profile in these patients.

### 3.4. Molecular Pharmacokinetic and Pharmacodynamic Changes of Statin Therapy Following Bariatric Surgery

Hydroxymethylglutaryl-coenzyme A (HMG-CoA) reductase inhibitors, or statins, form the most notable and widespread hypolipemic drug group, lowering LDL-c and TG concentrations while increasing HDL-c concentrations. The most prescribed drugs from this group are atorvastatin and rosuvastatin. Absorption is faster for atorvastatin due to its lipophilic properties; it is completely absorbed after oral administration but undergoes extensive first-pass metabolism in addition to being a P-gp substrate; hence, the bioavailability is about 12%. Rosuvastatin belongs to a hydrophilic subgroup of statins, and bioavailability after oral administration is 20% [79]. CYP3A4 plays a crucial role in the metabolism of atorvastatin, lovastatin, and simvastatin, while rosuvastatin is a substrate for (to a lesser degree) CYP2C9 and CYP2C19 enzymes. In a smaller percentage, metabolism takes place via the uridine diphosphate glucuronosyltransferase 1A1 (UGT1A1) and UGT1A3 routes.

The adverse effects of statin overdosing include statin-associated muscle symptoms (SAMSs), new-onset T2DM, neurological and neurocognitive impairment, hepatotoxicity and renal toxicity [80]. SAMSs, the most common of these effects, can be clinically presented as myalgia, myopathy, myositis with elevated creatinine kinase, or rhabdomyolysis, usually bilaterally affecting the large proximal muscle group of the lower extremities [81]. The prevalence of SAMSs varies among different statin classes, with the highest risk linked to statin drugs with lipophilic properties, such as simvastatin, atorvastatin, and lovastatin. Lipophilic statins use their ability to non-selectively diffuse into extrahepatic tissues like skeletal muscle [82]. There are many known risk factors for SAMSs development, such as female sex and a history of myopathy with other lipid-lowering therapy or high-dose statin therapy. Some studies indicate that as much as 60% of SAMS cases could be attributed to the concomitant use of statins and medications metabolized by the same hepatic cytochrome P450 enzymes [83]. The SAMS clinical index guidelines developed by the National Lipid Association provide a powerful tool for diagnosing and managing SAMSs in clinical practice. Due to specific risk factors and the fact that SAMSs account for 72% of adverse effects, adequate monitoring of bariatric patients is crucial [80].

The Biopharmaceutics Classification System (BCS) was thought to help in the prediction of oral drug exposure outcomes after bariatric surgery. The drugs in the BCS are classified into four classes based on solubility, permeability, and dissolution. Statins belong to class II in the BCS, which means that they are poorly soluble with low oral bioavailability but high permeability [84,85]. Although an assessment of physicochemical features based on the BCS alone is not adequate to clarify observed trends in modified oral drug bioavailability following bariatric surgery, available data suggest solubility plays an important role. In research conducted by Darwich et al., drugs classified as “solubility limited” displayed an overall reduction compared with “freely soluble” compounds, as well as an unaltered and increased post/pre-surgery oral drug exposure ratio [86]. Throughout different studies, the results for atorvastatin reveal significant differences in changes in oral bioavailability. The most varied outcomes in its systemic exposure were observed in a pharmacokinetic study of 12 participating patients in which the AUC ranged from a triple increase to a double decrease pre- and 3 to 8 weeks post-RYGB [87]. In previous reports, medications with the same physicochemical characteristics and additional substrates of CYP3A4/P-gp as atorvastatin (e.g., tacrolimus and cyclosporin A) showed a decrease in bioavailability after RYGB, so that an increase was a rather surprising result [88]. The explanation for this variability is not exactly clear, as it could be attributed to multiple factors, for example, insufficient number of respondents in the study, formulation effects, body weight of patients, time elapsed since surgery, CYP and P-gp genotypes, and bypass of bile salts [87,89]. An additional study by the same author demonstrated that the systemic exposure to atorvastatin is even more increased after BPD-DS, as the bypass in this surgical procedure is even longer [90]. The mean AUC proved to be twofold higher after surgery.

The most significant mechanism for the altered bioavailability of atorvastatin is hypothesized to be the bypass of the most metabolically active section of the intestines, even more noticeable with BPD-DS, given that the jejunum is fully bypassed in this procedure. However, this finding of increased bioavailability may seem counterintuitive, considering a decrease in the available surface area, shortened transit time frame for absorption, and subsequently reduced drug exposure [90]. Such outcomes can be explained by proximal intestinal metabolism restriction (i.e., a reduction in first-pass CYP metabolism in the small intestine) being a more significant factor than the minor surface area [90,91,92].

**Figure 2:**
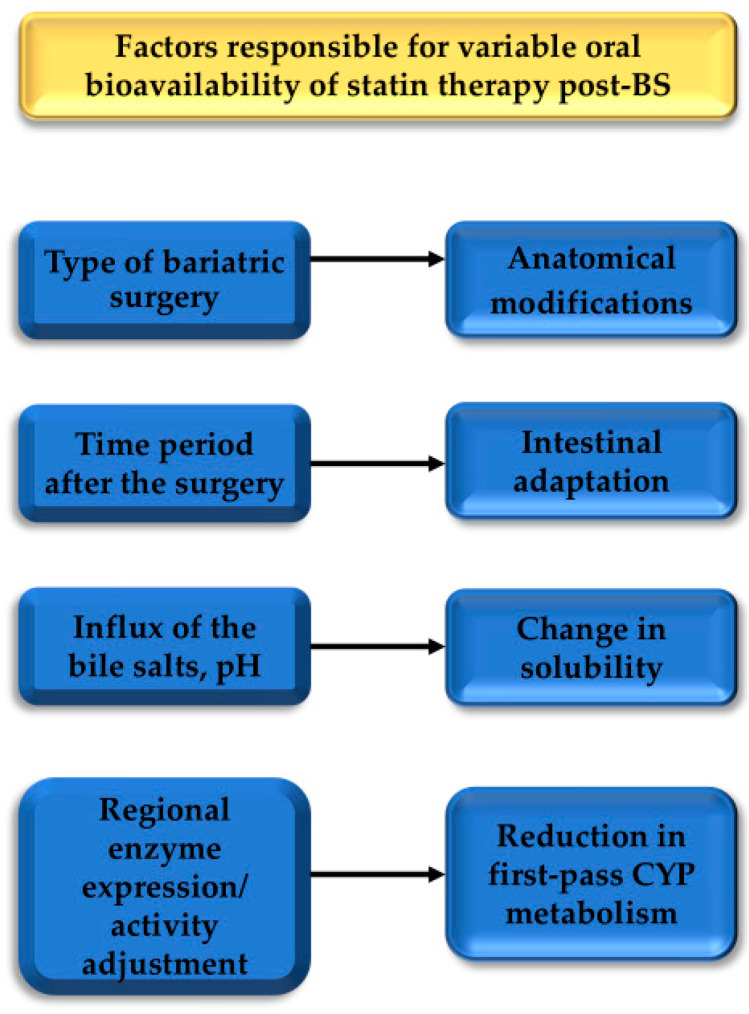
Summary of factors contributing to modified oral bioavailability of statin therapy following bariatric surgery. BS: bariatric surgery.

The process of weight loss after surgery is not permanent. It usually continues for about two years postoperatively, after which the weight either stabilizes or a small long-term weight gain is triggered [93]. A partial explanation lies in the changed habits of the patient, together with alterations in the intestines and metabolic adaptation. Similar mechanisms may also affect drug bioavailability. A long-term investigation study by Jakobsen et al. involved 20 patients, with a median of 27 months after surgery [94]. The initial AUC increase seen in the majority of patients 3–8 weeks after surgery was normalized long term, with more than half of RYGB patients and two-thirds of BPD-DS patients having decreased AUC compared with preoperative values. Although the small number of respondents in this study limits the generalization of its conclusions, these results proved that time is the most important factor. Interpretation of such a long-term outcome may be an intestinal adaptation that occurs over time. The basis of this compensatory mechanism is intestinal morphological and functional changes: villous hypertrophy, especially in the ileum, upregulation of enzymatic activity, and a possible increase in CYP3A protein expression [95,96]. The main factors contributing to the altered pharmacokinetics of statin therapy after bariatric surgery are illustrated in Figure 2.

A prospective pharmacodynamics and pharmacokinetic study on three statins (atorvastatin, rosuvastatin, and simvastatin) was performed by El-Zailik et al. [97]. The study showed a trend of decreased plasma concentration exposures for atorvastatin and rosuvastatin by three months and up to six months post-RYGB. As previously noted, rosuvastatin, unlike atorvastatin, is not mainly metabolized by CYP3A4. Rather, it is a substrate for multiple transporters expressed along the small intestine, with OAT2B1 and OAT1B1 being the most important of these [98]. In the mentioned study, only simvastatin showed an increasing trend in mean plasma concentration 6 months after RYGB, declining to pre-operative levels one year post-surgery. Simvastatin shares many similar characteristics with atorvastatin in lipophilicity and as a substrate for CYP3A4 enzyme and P-gp. The rising trend of simvastatin post-surgery could be interpreted through an increase in gastric pH after RYGB. Specifically, simvastatin is a lactone prodrug enzymatically hydrolyzed to active hydroxy acid form in the liver to achieve pharmacological activity [99]. The conversion to the active acid form acts pH-dependently [97,100]. For samples of hydroxy acid and lactone forms, maintaining the pH of the solution around 4–5 minimizes interconversion. Increasing the pH above 6 facilitates the conversion of lactone to acid, whereas lowering the pH enables the conversion from acid to lactone or lactone to acid in the non-ionized form [101,102]. These results could also be connected to differences between the half-life length of these three statin drugs in correlation with sampling time due to the shorter half-life of simvastatin compared to atorvastatin and rosuvastatin (2 vs. 14 and 19 h). The return of plasma levels one year post-RYGB to baseline levels implies an adaptation of the gastrointestinal tract.

Despite the available research, there is still a lack of adequate data for statin effectiveness post-bariatric surgery, as well as for the other drug groups. Because of this deficiency in clinical data, some researchers have tried a simulation on an in vitro mechanistic model as an instrument for further examination [103,104]. The advanced dissolution absorption and metabolism (ADAM) model was constructed to examine the validity of predicting the influence of surgery on oral bioavailability through a designed figure of the small intestine based on normal intestinal physiology [104]. Developed models for RYGB and BPD-DS included specific anatomical and physiological parameters that are modified post-bariatric surgery: stomach capacity, gastric emptying time, small intestinal transit time, pH value of the stomach and intestines, the influx of bile salts, and adjustment in the regional expression of drug-metabolizing enzymes and efflux transporters, predominantly P-gp. This mechanistic stimulation also incorporates the biochemical characteristics of the drugs, contributing to the complexity of the issue: solubility, permeability, dissolution, gut wall metabolism, and dosage level. The obtained data for oral bioavailability of atorvastatin post-surgery were highly reflective of the previously observed trend. The reduction of the portion of dose absorbed across the intestinal wall, or, in other words, the increased portion of drug dose evading intestinal metabolism, was declared to be of the most importance when considering the overall effect on oral bioavailability. The ADAM model was not able to recapture the observed twofold increase in atorvastatin AUC post-BPD-DS, emphasizing that additional physiological alterations have yet to be explored.

### 3.5. Future Directions

Progressively with the weight loss, normalization or significant reduction of the lipid-profile value is expected. Periodic monitoring is important, approximately every 3–6 months, until weight loss stabilizes. Previous recommendations suggested that, if needed, lipid-lowering agents should be discontinued [40]. Nonetheless, just as weight loss takes place over a limited period, discontinuation of statin therapy can lead to a rebound phenomenon in the lipid profile as well, which is often observed. Hence, based on the review of the currently available literature, a better approach for physicians in clinical practice would be to reduce the statin dose to the lowest effective, e.g., half of the dose post-surgery, with continuous monitoring of lipid levels (Table 2) [97]. Cautious follow-up enables rational dose corrections to achieve a therapeutic window without adverse effects or, on the other hand, subtherapeutic responses.

Today, when obesity has become a public health problem and an escalating global pandemic, the number of developed and available therapeutic options has increased—from conservative treatment methods to the operating room. Further research is needed that focuses on the correlation of obesity with the anatomical and physiological changes arising from bariatric surgical treatment. Some of these occur early, and some with a time lag after bariatric treatment. All these factors need to be taken into consideration when developing follow-up protocols for bariatric patients and providing adequate care. To achieve these goals, well-conceived longitudinal research is necessary, considering obesity, weight loss per se, comorbidities, and the features of each type of bariatric surgery procedure with their consequent anatomical and physiological changes of the body and later adaptive processes.

## 4. Conclusions

Currently available research has demonstrated high inter- and intravariability in the adaptive mechanisms and sustainable different values of pharmacokinetic parameters after administering the same dose of certain drugs. However, because of the extensive multifactorial nature of this issue, there are neither guidelines nor a single, unique model that can be followed in clinical practice. The different effects of bariatric surgery on the pharmacokinetics of statins underscore the critical need for a personalized approach as an essential strategy in the management of dyslipidemia in post-bariatric surgery patients. Therefore, the recommendations would include continuous monitoring of the lipid profile every 3–6 months post-BS, along with the continuation of statin therapy. Before surgery, the dose should be reduced to the lowest effective, usually half of the dose prescribed pre-surgery. Monitoring allows dose titration depending on lipid-lipoprotein values, with no need for rapid dose adjustment post-BS. Regular follow-ups and pharmacokinetic assessments can help in adjusting the therapy to achieve the desired therapeutic outcomes while minimizing adverse effects. Future research should continue to explore the mechanisms behind the variability in statin pharmacokinetics post-surgery, and create specific clinical recommendations to enable individualized treatment options in this growing patient population.

## Figures and Tables

**Table 1:** Anatomical and physiological alterations following restrictive and malabsorptive types of bariatric surgery with potential pharmacokinetic implications after oral administration (according to ref. [39,40,41]).

Anatomical/Physiological Alterations Post-BS	Type of Procedure		Potential Pharmacokinetic Implications for Oral Dosage Form
Reduced gastric capacity		Restrictive	Decreased dissolution, disintegration Change in the C_max_ and T_max_
Accelerated gastric emptying time		Restrictive	
Increased gastric pH	Malabsorptive	Restrictive	Decreased basic drug solubility Increased acidic drug solubility
Reduced absorptive surface area in the small intestine	Malabsorptive		Decreased dissolution, absorption
Decreased exposure to metabolizing enzymes	Malabsorptive		Decreased first-pass metabolism (especially CYP3A4 substrates)
Decreased exposure to carrier proteins	Malabsorptive		Variable absorption (influx transporters/efflux pumps)
Restricted enterohepatic circulation	Malabsorptive		Decreased lipophilic drug dissolution and absorption
Dissociation of bile salt flow	Malabsorptive		
Decreased intestinal transit time	Malabsorptive		Incomplete dissolution Change in the C_max_ and T_max_
Increased gastrointestinal pH	Malabsorptive	+	Change in the C_max,_ T_max,_ AUC

BS: bariatric surgery; C_max_: the maximum concentration of a drug; T_max_: time to peak drug concentration; CY: cytochrome; AUC: area under the curve.

**Table 2:** An overview of the available literature on pharmacokinetic changes of statin therapy after bariatric surgery.

Drug	Dosage Form	BS Technique	*N*	Follow-Up (Post-BS)	Pharmacokinetic Impact (AUC)
Atorvastatin	Oral	RYGB	12	3–8 weeks	Threefold increase/twofold decrease [83]
Atorvastatin	Oral	BPD	10	4–8 weeks	Twofold increase [86]
Atorvastatin	Oral	RYGB, BPD	20	21–45 months	Long-term normalization/ decrease [90]
Atorvastatin	Oral	RYGB	3	3 and 6 months	Decrease of 58% at 3 months, 75% at 6 months [93]
Rosuvastatin	Oral	RYGB	4	3 and 6 months	Decrease of 43% at 3 months, 61% at 6 months [93]
Simvastatin	Oral	RYGB	5	3, 6 and 12 months	Increase of 33% and 150% at 3 months, doubled at 6 months; decline to pre-operative levels at 1 year [93]

AUC: area under the curve; BS: bariatric surgery; *N*: number of participants.

## Data Availability

The data presented in this study are available upon request from the corresponding author.

## Abbreviations

WHO	World Health Organization
BMI	body mass index
T2DM	type II diabetes mellitus
US	United States
FDA	The Food and Drug Administration
SG	sleeve gastrectomy
RYGB	roux-en-Y gastric bypass
LAGB	laparoscopic adjustable gastric banding
BDP-DS	biliopancreatic diversion with duodenal switch
P/D1	programmed cell death protein 1
AUC	area under the curve
CYP	cytochrome P450
UGT	uridine diphosphate glucuronosyltransferase
P-gp	P-glycoprotein
GLP-1	glucagon-like peptide-1
GIP	glucose-dependent insulinotropic polypeptide
TG	triglyceride
HDL-c	high-density lipoprotein cholesterol
LDL-c	low-density-lipoprotein cholesterol
CVR	cardiovascular risk
HMG-CoA	hydroxymethylglutaryl-CoA
SAMs	statin-associated muscle symptoms
BCS	The Biopharmaceutics Classification System
ADAM	The Advanced Dissolution Absorption and Metabolism
